# Sex: the last real binary?

**DOI:** 10.1530/RAF-24-0005

**Published:** 2024-04-12

**Authors:** Shiuli Bhattacharyya

**Affiliations:** 1 UCL, Department of Medical School, London, UK

**Keywords:** reproduction, sex determination, gender, fertility

## Abstract

The rights of transgender and intersex people have become a contentious issue in our current political climate. Whether it be the rights of intersex athletes such as Caster Semenya (who identifies as a woman) to compete in elite sport, or the rights of transgender women to use women’s only spaces, there is an increasingly fierce debate as to the legitimacy of people’s gender and sexual identities and what parameters should be used to define them. A common argument accepted by most in our society is that while gender may be a spectrum, sex is an inalienable binary.

The rights of transgender and intersex people have become a contentious issue in our current political climate. Whether it be the rights of intersex athletes such as Caster Semenya (who identifies as a woman) to compete in elite sport (Pielke *et al.* 2019), or the rights of transgender women to use women’s only spaces (Bagagli *et al.* 2021), there is an increasingly fierce debate as to the legitimacy of people’s gender and sexual identities and what parameters should be used to define them. A common argument accepted by most in our society is that while gender may be a spectrum, sex is an inalienable binary. An example of how prevalent this idea is seen in British prime minister Rishi Sunak’s recent Conservative party conference speech in which he asserted that: ‘We shouldn’t get bullied into believing that people can be any sex they want to be. They can’t. A man is a man, and a woman is a woman – that’s just common sense’ (https://www.washingtonpost.com/world/2023/10/05/rishi-sunak-gender-identity-transgender/ (accessed 25 October 2023)).

This idea is especially prevalent online, with many, including prominent figures ranging from right-wing politicians to radical feminists, engaging in debates as to what constitutes a ‘real woman’ (Montiel-McCann 2022). Often, this leads to womanhood being defined through narrow definitions such as the presence of a vagina, (https://www.independent.co.uk/news/uk/politics/wes-streeting-trans-rights-women-penis-b2047117.html (accessed 25 February 2023)), menstrual periods (https://www.independent.co.uk/life-style/jk-rowling-tweet-women-menstruate-people-transphobia twitter-a9552866.html (accessed 25 October 2023)), or chromosomal sex (https://talk.tv/news/27326/lesbians-are-being-erased-and-harmed-by-an-ideology-but-it-isnt-gender-identity freda-wallace (accessed 25 October 2023)).

In this article, I will argue that this is an overly simplistic explanation of sex differentiation, which excludes intersex people and individuals with phenotypic characteristics that vary from their sex assigned at birth, such as women with PCOS. I will do so by drawing on historical and contemporary examples and arguing that a strict sex binary is scientifically inaccurate and exclusionary.

Sex determination at the chromosomal level has historically been defined by the presence or absence of the SRY gene on the Y chromosome, which promotes the development of testes and therefore an anatomically ‘male’ fetus (Epplen *et al.* 1981). This criterion formed the basis of so-called sex testing at the 1992 and 1996 Olympic Games, whereby all female athletes were required to undergo SRY gene testing (https://www.nytimes.com/2016/07/03/magazine/the-humiliating-practice-of-sex-testing-female-athletes.html (accessed 25 October 2023)).

In subsequent games, this strategy was abandoned as it became clear that chromosomal sex alone was not enough to determine ‘female’ athletic ability. In the 1996 Olympics, eight athletes were initially barred from competing due to the presence of a Y chromosome but were ultimately cleared as they all had androgen insensitivity (Dickinson *et al.* 2002), meaning despite being genetically male and producing high levels of testosterone, a loss of function mutation in the androgen receptors meant that their bodies did not use this testosterone to produce a typically male phenotype (Hughes *et al.* 2012). Instead, the testosterone produced (in this condition) is aromatised into oestrogens, leading to the development of female secondary sexual characteristics (Hughes *et al.* 2012). In essence, the Olympic committee conceded that these athletes were women, despite their genetic sex. Other Disorders of Sexual Development (DSD) involve atypical chromosomal sex itself and result in primary hypogonadism (García-Acero *et al.* 2020). In Klinefelter’s syndrome, for example, the most common DSD condition, the fetus will have a 47 XXY karyotype due to a meiotic non-dysfunction error which results in the addition of an extra X chromosome (in some cases further copies of the X chromosome may be added leading to 48 XXXY, 49 XXXXY, etc.) (Los & Ford 2023). The presence of an extra X chromosome leads to primary testicular failure (underdevelopment of the testis leading to much reduced testosterone production) which leads to symptoms such as infertility and gynaecomastia (Los & Ford 2023) (Fukami 2023). Despite this, a person with Klinefelter’s syndrome is generally phenotypically male and is identified as such. A person with Turner’s syndrome (45 X0 karyotype) will similarly be deemed female despite experiencing ovarian dysfunction and primary amenorrhoea (Fukami 2023). Chromosomes alone are not sufficient in determining sex in every case.

Labour MP Wes Streeting claimed in 2022 on a popular radio show that: ‘Women have vaginas and men have penises, here ends my biology lesson’ (https://www.independent.co.uk/news/uk/politics/wes-streeting-trans-rights-women-penis-b2047117.html (accessed 25 October 2023)).

This argument is echoed across the internet and is also often posed as simple biology. Similar sentiments have been echoed in the past. In fact, prior to the varying forms of genetic testing that were introduced in the Olympics since the late 1960s, female athletes whose sex was in question were subjected to genital exams before competing (Rupert 2016). This practice was not only demeaning but inaccurate. Whilst it is undeniable that most women have vaginas and most men have penises, this is not always the case. Several conditions may result in ambiguous genitalia, including congenital adrenal hyperplasia (CAH) (Yau *et al.* 2022). The most common cause of ambiguous genitalia is 21-hydroxylase enzyme deficiency (White & Speiser 2000). The deficiency of this enzyme leads to an increase in 17-dehydroxyprogesterone, which is converted to testosterone through a series of reactions (Yau *et al.* 2022). This prenatal exposure to androgens in a 46 XX fetus can lead to enlargement of the clitoris and labial fusion, leading to ambiguous genitalia at birth (White & Speiser 2000). Despite these babies being genetically female, having normal internal genitalia (ovaries, fallopian tubes, and uterus), and often normal reproductive function, by this logic, they would be classified as male.

Higher levels of testosterone are typically associated with men, while higher levels of oestrogen are typically associated with females. We have already discussed examples where this is not the case, but some may argue that these are fringe and not representative. However, in several much more common conditions, circulating testosterone and oestrogen levels may not match the typical male or female pattern. PCOS is the most common endocrinopathy in women of reproductive age, with some estimates suggesting that up to 20% of women may have the condition (Teede *et al.* 2010). While clinical presentation varies, many women suffer from hyperandrogenic symptoms due to a few complex and poorly understood mechanisms (Behboodi Moghadam *et al.* 2018). This often results in male pattern hair growth and subfertility as well as biochemical increases in testosterone and other androgens (Teede *et al.* 2018). Other conditions such as Cushing’s syndrome or corticosteroid use can also cause hyperandrogenism (Arnaldi & Martino 2019). These symptoms can cause deep distress for sufferers in part due to not meeting social expectations of womanhood (Khomami *et al.* 2015).

Prominent feminists such as Germaine Greer have made comments about transgender women which may also be painful for cisgender women. In a 1989 article written in the Independent, Greer describes an encounter she had with a transgender woman: ‘I smirked and nodded and stepped backwards, trying to extricate my hand from the enormous, knuckly, hairy, be-ringed paw that clutched it’ (https://www.vice.com/en/article/yvxxyy/germaine-greer-paris-lees-hypocrisy-left-free-speech (accessed 25 October 2023)).

The following quote from a large qualitative study of women with PCOS is significant: ‘It’s bad for a woman to have a beard like the men; it’s really bad; well, this is really embarrassing to be hairy and have a beard’ (Nasiri Amiri *et al.* 2014).

The rhetoric surrounding sex and gender in the media is, of course, harmful to trans women, a marginalised group with high rates of suicide and poorer quality of life (Bailey *et al.* 2014), but also to cisgender women whose phenotype does not perfectly match their gender. Whilst there are legitimate concerns on either side of the debate, in my opinion, binary definitions of biological sex based on anatomy, chromosomes, or hormones in isolation are doomed to be inaccurate, as demonstrated by the cases discussed in this article. DSD conditions are rare, but a binary by definition only has two options, and as I have hopefully demonstrated, there are outliers. The existence of intersex people cannot be debated, and as a highly stigmatised minority, their presence must be acknowledged in these conversations. A more nuanced perspective that appreciates the different layers of sex differentiation holistically and acknowledges some variation is therefore more scientifically accurate and less divisive.

## Declaration of interest

The author declares that there is no conflict of interest that could be perceived as prejudicing the impartiality of this article.

## Funding

This work did not receive any specific grant from any funding agency in the public, commercial, or not-for-profit sector.
